# TRIM21 Neutralizes Hazara Virus Using a Dual Mechanism of Nucleoprotein Caging and Ubiquitination

**DOI:** 10.3390/v18090974

**Published:** 2026-09-04

**Authors:** Aminu S. Jahun, Boglarka A. Vamos, Anna Albecka, Marina Vaysburd, David Hawman, Leo C. James

**Affiliations:** 1 MRC Laboratory of Molecular Biology, Francis Crick Avenue, Cambridge CB2 0QH, UK; ajahun@mrclmb.ac.uk (A.S.J.);; 2 Laboratory of Viral Zoonoses, Integrated Research Facility Frederick, NIAID/NIH, Frederick, MD 21702, USA

**Keywords:** TRIM21, Hazara virus, CCHFV, antibody, nucleoprotein, antiviral immunity, neutralization, EDNA, ADIN, caging

## Abstract

Crimean–Congo Hemorrhagic Fever Virus (CCHFV) is a tick-borne bunyavirus with widespread and growing geographic distribution that causes severe hemorrhagic fever and death. Vaccine candidates targeting the viral nucleoprotein (NP) have shown efficacy in both mouse and non-human primate models but the mechanism of protection is unclear. Here we employ the closely related Hazara virus (HAZV) to investigate how the intracellular antibody receptor TRIM21 uses anti-NP antibodies to neutralize infection. We show that TRIM21 can detect incoming NP particles within hours of infection and that this results in a potent block to infection. Electroporated-antibody-dependent neutralization assay (EDNA) experiments reveal that TRIM21 inhibits viral transcription, protein expression and genome synthesis and reduces the production of infectious virions. Mutations and domain deletions within TRIM21 reveal that neutralization requires antibody-binding by the PRYSPRY domain but is only partially dependent on the E3 ubiquitin ligase RING domain. The data suggest a dual restriction mechanism in which NP cross-linking by TRIM21 physically interferes with NP function whilst parallel ubiquitination labels the protein for degradation. This dual mechanism is similar to that used by TRIM5 against retroviruses and suggests that antiviral TRIMs may utilize their capacity for self-assembly both for catalytic activation and viral caging.

## 1. Introduction

Crimean–Congo Hemorrhagic Fever Virus (CCHFV) is a tick-borne bunyavirus endemic to parts of Europe, Africa and Asia and with an expanding geographic range due to climate change. CCHFV is listed by the WHO as one of their top 10 priorities for emerging virus research due to its high mortality rate (30%) and difficulties in treatment and prevention. There are currently no approved vaccines but several are in human clinical trials. Interestingly, multiple pre-clinical studies have shown that vaccines expressing the CCHFV nucleoprotein (NP) can confer remarkable protection in mice and non-human primates (NHPs). A candidate DNA-based vaccine based on both glycoprotein (Gc) and nucleoprotein (NP) prevented CCHFV infection in NHPs [1]. This vaccine induced a strong anti-NP response but no significant Gc-specific antibodies, suggesting that protection was primarily mediated by anti-NP antibodies [1]. NHPs vaccinated with a plasmid expressing NP alone were also protected against challenge [2]. Subsequent works evaluating multiple vaccine platforms including messenger RNA (mRNA), self-amplifying RNA, subunit, and virally vectored vaccines have demonstrated protection in mice due to responses against NP [3,4,5,6,7,8], whilst passive transfer of an anti-NP monoclonal antibody (mAb) was sufficient to confer protection [9]. A self-replicating mRNA vaccine based solely on NP has since been developed which protects against CCHFV infection in both NHPs and mice [10,11]. However, protection was unexpectedly found to be independent of Fc gamma receptors (FcγRs), complement component 3 (C3), natural killer cells and CD4 and CD8 T cells [11]—a finding consistent with results from anti-NP mAb passive transfer experiments where protection was also maintained in both Fcγ and C3 knockouts [9]. Notably, vaccine protection was only lost in animals knocked out for the antibody receptor tripartite motif-containing protein 21 (TRIM21). Passive transfer of sera from vaccinated animals protected wildtype but not TRIM21-deficient mice against CCHFV challenge. Sera from CCHFV-exposed humans could also inhibit CCHFV replication in a TRIM21-dependent manner, suggesting that this mechanism is not restricted to vaccine-mediated responses [11]. Together these data suggest that antibodies against the CCHFV NP can confer protection against CCHFV in a TRIM21-dependent manner.

TRIM21 is a highly conserved, high-affinity antibody receptor that is uniquely expressed in the cytosol of most cells in mammals [12]. TRIM21 intercepts incoming antibody-coated pathogens, both viruses and bacteria, and targets them for degradation, thus blocking infection [13,14]. TRIM21 is a member of the tripartite motif family and is a multidomain protein consisting of RING, B Box, coiled-coil and PRYSPRY domains. TRIM21 uses its C-terminal PRYSPRY domain to bind antibodies by recognizing the ‘HNHY’ motif at the end of CH3 [12]. Importantly, this motif is different from, and structurally distant to, the binding site recognized by FcγRs. The coiled-coil domain is responsible for dimerizing TRIM21, allowing binding of both heavy chains on immunoglobulin G (IgG) simultaneously [12]. The TRIM21 RING domain is an E3 ubiquitin ligase whose activity is tightly regulated by the B Box domain, which occupies the E2 binding site by the two RINGs being held apart at either end of the dimerizing coiled-coil [15,16]. TRIM21 ubiquitination is activated by a substrate-induced clustering mechanism, which brings RING domains into proximity allowing them to dimerise and accept a ubiquitin-charged E2 [15,16]. Since its original discovery in the context of adenovirus neutralization, TRIM21 has been shown to be highly promiscuous in its substrate specificity and capable of ubiquitinating and degrading a wide range of substrates in membranes, in the cytosol and in the nucleus and ranging in size from small protein oligomers (e.g., IκB kinase ) [17], to large protein aggregates (e.g., tau) [18] and bacteria (e.g., Salmonella) [14].

Enveloped viruses were initially thought to escape TRIM21-mediated restriction because antibodies bound to the surface of viral membranes are left in the lumen of the endosome upon receptor-mediated endocytosis. Nevertheless, non-neutralizing anti-NP antibodies were found to protect against lymphocytic choriomeningitis virus (LCMV) infection in mice TRIM21-dependently, by promoting antigen presentation and eliciting a strong epitope-specific cytotoxic T cell response [19]. However, this mechanism cannot explain the TRIM21-dependent protection observed in CCHFV [11] or the similar vaccine protection observed for severe fever with thrombocytopenia syndrome virus [20] as there was no requirement for T cells for either. Previously, we developed an in vitro assay called EDNA, which stands for ‘electroporated-antibody-dependent neutralization assay’, that allows the activity of non-neutralizing antibodies to be studied in vitro by directly delivering them into the cytosol [21]. EDNA experiments have shown that non-neutralizing anti-nucleoprotein antibodies against severe acute respiratory syndrome coronavirus 2 (SARS-CoV-2) or mouse hepatitis virus (MHV) are capable of blocking infection by recruiting TRIM21 and mediating NP degradation [21]. We decided to employ the EDNA assay to investigate the TRIM21-dependent mechanism of CCHFV vaccine protection using Hazara virus (HAZV), a closely related *Orthonairovirus* and commonly used model for CCHFV, which is widely used at a lower biocontainment level unlike CCHFV. Anti-NP antibodies have been shown to induce cross-reactive protection against CCHFV and HAZV in multiple species highlighting significant similarities between the viruses [22]. Here we show that TRIM21 can use anti-NP antibodies to potently block HAZV infection by halting viral transcription, protein synthesis and the production of new virions. Unexpectedly, the data suggest that TRIM21-dependent neutralization occurs through two parallel and independent processes—the physical caging of antibody-bound NP by clustered TRIM21 and RING-dependent NP ubiquitination.

## 2. Methods

### 2.1. Cell Lines

SW13, BSR-T7, L929, HEK293T, and HeLa cells were grown in high glucose Dulbecco’s Modified Eagle Medium (DMEM, Thermofisher, Waltham, MA, USA, #11965092) containing L-glutamine and supplemented with 10% heat-inactivated fetal cow serum (HyClone, Logan, UT, USA, #SH30084.03) and penicillin/streptomycin, at 37 °C in the presence of 5% CO2. Generation of the TRIM21-KO HeLa cells [23], TRIM21-KO L929 cells [21] and TRIM21-KO HEK293T cells reconstituted with FusionRed TRIM21 [14] was previously described. L929 (product no. CCL-1) and HeLa cells (product no. CCL-2) were purchased from ATCC, Manassas, VA, USA. HEK293T cells were a kind gift from Dr Ravi Gupta (University of Cambridge, Cambridge, UK). SW13 and BSR-T7 cells were a kind gift from Dr John Barr (University of Leeds, Leeds, UK).

### 2.2. Virus Reverse Genetics

Virus rescue was carried out according to a previously described protocol [24]. Briefly, BSR-T7 cells were seeded in 6-well plates overnight and transfected with 1.2 µg each of the T7 promoter-driven S, M, and L segment rescue plasmids and a 0.6 µg of T7 polymerase-expressing plasmid (pCAG-T7pol, Addgene #59926), using the TransIT-LT1 transfection reagent (Mirus Bio, Madison, WI, USA, #MIR2300). The media was replaced 24 h post-transfection and the virus is harvested 4 days later. To generate higher titre virus stocks, SW13 cells pre-treated with 10µM of Ruxolitinib (Selleckchem, Houston, TX, USA, #S1373) were incubated with HAZV at 37 °C for 1 h, after which the Ruxolitinib-containing media was removed. The cells were washed twice with complete DMEM and grown in fresh media for 2–5 days.

To rescue HAZV N-WT, pMK-RQ-S-mScarlet, pMK-RQ-M and pMK-RQ-L were used for the S, M, and L segments, respectively. pMK-RQ-S-mScarlet_N-HA and pMK-RQ-S-mScarlet_N-ALFA were used as the S segment rescue plasmids for HAZV N-HA and HAZV N-ALFA, respectively. The pMK-RQ-S-eGFP, pMK-RQ-M and pMK-RQ-L rescue plasmids were a generous gift from Dr John Barr (University of Leeds, Leeds, UK).

### 2.3. Virus Infection

Unless otherwise indicated, cells were infected with HAZV by incubating adherent cell monolayers with virus inoculum at 37 °C for 1–2 h, after which the inoculum is replaced with fresh media. For virus growth curves and confocal imaging assays, the cells were spinfected at 4 °C by centrifugation at 380× *g* for 1–2 h followed by media replacement. The cells were then transferred to the incubator or the Incucyte (Sartorius, Göttingen, Germany).

### 2.4. Inhibitor Treatment

Adherent cell monolayers were treated with indicated concentrations of bafilomycin (Tocris, Avonmouth, Bristol, UK, #1334), TAK243 (Selleckbiochem, Houston, TX, USA, #S8341), MG132 (Sigma Aldrich, St. Louis, MO, USA, C2211), epoxomicin, or an equivalent amount (*v*/*v*) of DMSO (Fisher scientific, #BP231-100) for 2 h prior to infection. The cells were incubated with the virus inoculum for 1 h, following which the inoculum was replaced by fresh inhibitor-containing media, and the cells were transferred to the Incucyte (Sartorius) for continuous live cell imaging.

### 2.5. EDNA

Electroporated-antibody-dependent neutralization assay (EDNA) was carried out using the Neon electroporation system (Thermofisher, Waltham, MA, USA) as previously described [21]. Briefly, cells were trypsinised, counted, washed once with PBS, and resuspended in PBS or buffer R. 12 µL of resuspended cells, containing the required number of cells, were transferred into a new tube, and 2 µL of antibody was added. The cell/antibody mixture was mixed gently and taken up using the 10 µL electroporation pipette tip, and the cells were electroporated at 1400 V, 20 ms and 2 pulses. Electroporated cells were then transferred into new tubes containing antibiotics-free media and seeded overnight, prior to infection with HAZV. Infection was subsequently monitored by live cell imaging in an Incucyte S3 (Sartorius, Göttingen, Germany), Western blotting or qPCR. Recombinant anti-HA (12CA5) monoclonal antibodies and anti-ALFA nanobody-Fc used for ENA assays here were produced by Genscript.

### 2.6. RNA Extraction

RNA was extracted using the RNeasy mini kit (Qiagen, Venlo, Netherlands, #74104), according to the manufacturer’s protocol. Briefly, samples lysed in buffer RT supplemented with dithiothreitol (DTT) were passed through gDNA removal columns, prior to addition of ethanol to 35% (*v*/*v*). The samples were then passed through the RNA columns, washed once with buffer RW1 and twice with buffer RPE. RNA samples were eluted in Milli-Q water.

### 2.7. RT-qPCR

For standard qPCR, reverse transcription was carried out using the M MLV RT kit (Promega, Madison, WI, USA, #) according to the manufacturer’s protocol. Briefly, the sample RNA was incubated with random hexamers (Roche, Basel, Switzerland, #11034731001D2) at 70 °C for 5 min and chilled in ice, followed by addition of the RT master mix (final concentration of 0.4 mM dNTPs, 1× reaction buffer, 0.5 µ/reaction RNaseOUT (Thermofisher, #10777019), and 0.5 µ/reaction M-MLV [Promega, Madison, WI, USA, #M1701]). The samples were then incubated first at 37 °C for 60 min and then 95 °C for 5 min. cDNA was diluted 1:4 in Milli-Q water and qPCR was carried out using 100 nM each of forward and reverse primers targeting the HAZV S segment, and a SYBR green mastermix containing a final concentration of 1× reaction buffer (Eurogentec RT-SN73-05NR), 2.5 mM MgCl2 (Eurogentec RT-SN73-05NR), 0.2 mM dNTPs (Thermofisher, #R0181), 1:20,000 SYBR Green (Molecular Probes S-7563), 0.5 M Betaine (Sigma, 61962), and 25 U/mL Gold Star polymerase (Eurogentec, Seraing, Liège, Belgium, RT-SN73-05NR). Samples were ran in a StepOne Plus qPCR machine (Thermofisher). The previously described HAZV S segment rescue plasmid (pMK-RQ-S) was used as a DNA standard for determining genome copy number. qPCR primer sequences are listed in Supplementary Table S1.

For strand-specific qPCR, reverse transcription was carried out with a virus-specific primer that targets the HAZV S segment and carries a non-viral tag sequence on the 5’ end. The reaction was performed using the SuperScript IV Reverse Transcriptase (Thermofisher, #18091050) at 65 °C for 10 min. qPCR was then carried out using the same protocol as the standard qPCR described above, except the primers used which include a virus-specific forward primer and a non-viral tag-only reverse primer. Single-stranded DNA used as strand-specific qPCR standards was synthesized by Integrated DNA Technologies. Fold difference between matched and mismatched templates/qPCR primers was calculated using the 2^−ΔΔCT^ method relative to mismatched conditions. Strand-specific primers and oligo sequences are listed in Supplementary Table S1.

### 2.8. Plasmids

Plasmids used here are listed in Supplementary Table S2. The EGFP sequence in the previously described HAZV S segment rescue plasmid pMK-RQ-S-eGFP [25] was switched with mScarlet using Gibson assembly. This new plasmid was used to rescue N-WT HAZV. For N-HA and N-ALFA HAZV, HA and ALFA tags were added to their c-termini, respectively, using Gibson assembly.

### 2.9. Lentivirus Production and Cell Transduction

The lentivirus production protocol used here was previously described [16]. Briefly, HEK293T were seeded in 10-cm dishes overnight, followed by co-transfection of 2 µg of expression plasmid, 2 µg of pCRV Gag-Pol, and 1 µg of pMDG.2, using 60 µL of lipofectamine 2000 (Thermofisher, # 11668019) per dish. The cells were incubated overnight, and the transfection media was replaced with fresh media the next day. The lentiviruses produced were harvested 24 h after the media change. For cell transduction, TRIM21-KO HeLa cells were seeded in 6-well plates overnight. The next day, the media was replaced with 2 mL of complete DMEM supplemented with 300 µL of crude lentivirus stock per well. The cells were then incubated at 37 °C for 2 days, before selection using 1 µg/mL of puromycin (Invivogen, San Diego, CA, USA, #ant-pr-1).

### 2.10. Western Blotting

Cells were washed once with PBS prior to incubation in NP-40 lysis buffer (50 mM of Tris-HCl (pH 7.4), 150 mM of NaCl, 1%NP-40 and 5 mM of EDTA) (Thermofisher, #J60766.AK) supplemented with Halt Protease/Phosphatase inhibitor cocktail (Thermofisher, #1861281) on ice for 15 min. Samples were homogenized by pipetting up and down, and clarified by centrifugation at 17,000× *g*, 4 °C, for 15 min. NuPAGE LDS Sample Buffer (Thermofisher, #NP0007) was added to the supernatant to 1×, followed by incubation at 95 °C for 5 min. Samples were then ran on a 4–12% NuPAGE Bis-Tris Protein Gel (Thermofisher, NP0321BOX), transferred onto nitrocellulose membranes using an iBlot 3 transfer system (Thermofisher), and incubated in 5% milk in PBST for 1 h at room temperature, followed by primary antibody at 4 °C overnight, and secondary antibody for 1 h at room temperature. Images were obtained using an Odessey CLx imager (LICOR bio, Lincoln, NE, USA). Primary antibodies used here are listed in Supplementary Table S3.

### 2.11. Confocal Microscopy

Epitope-tagged NP expression was compared to WT by infecting 293T cells with N-HA, N-ALFA or wildtype HAZV. Cells were fixed 24 h post-infection with 4% formaldehyde and permeabilised with 0.5% Triton X-100 (Sigma-Aldrich). Wells were blocked using 3% FBS in PBS then subsequentially incubated with primary and secondary antibodies for 1 h each at room temperature with multiple washes between incubations. TRIM21-knockout HEK293T cells reconstituted with FusionRed TRIM21 were used to observe TRIM21 recruitment to antibody-coated NP and ubiquitination. Cells were electroporated with wildtype or H433A mutant human anti-HA antibodies and cold spinfected the next day with N-HA HAZV. Cells were fixed and stained 2 h post-infection as above. Primary antibodies used were: anti-NP mouse serum, anti-NP rabbit serum, rabbit HA-Tag (Cell Signaling Technology, Danvers, MA, USA, 3724), rabbit anti-ALFA serum (NanoTag Biotechnologies, Göttingen, Germany, N1580), and polyubiquitin FK2 (Enzo Life Sciences, Farmingdale, NY, USA, BML-PW8810). Secondary AlexaFluor conjugated antibodies are all from Invitrogen. Nuclei were stained with Hoechst 33342 (Abcam, Cambridge, UK, ab228551). All images were acquired on a Nikon W1 spinning disk inverted microscope with 10×/1.4NA oil objective and sCMOS camera. For any downstream analysis at least five images of each condition were taken. The proximity of puncta in several channels was evaluated using custom-made macros in ImageJ [14]. Within each experiment, images were acquired with identical instrument settings and batch-processed together.

### 2.12. Split-Luciferase TRIM21 In Vitro Assay

To determine if TRIM21 could bind via antibody to His-HAZV NP, a split-luciferase assay was used involving recombinantly expressed His-lipoyl-SmBit-TRIM21 and His-lipoyl-LgBit-TRIM21 proteins. His-HAZV NP and HIV nucleocapsid (NC) as a negative control were also recombinantly expressed and purified. A master mix containing 50 mM of SmBit and 50 mM of LgBit-TRIM21 in buffer with PBS and 1 mM of TCEP with 5% final concentration of albumin was mixed with 2 μM of either HAZV NP or HIV NC. Serial 4-fold dilution of anti-NP sheep serum (kind gift from Dr John Barr, University of Leeds, Leeds, UK) was carried out in PBS with 1 mM of TCEP. 20 μL per serum dilution was mixed with 5 μL of master mix in duplicate and incubated for 20 min at room temperature. Per reaction 20 μL of Nano-Glo substrate (Promega) diluted in PBS was added and incubated for another 10 min while shaking. Luminescence was measured using GloMax Explorer Multimode Microplate Reader (Promega).

### 2.13. Data Analysis

Data was analyzed using Microsoft Excel (Microsoft, Redmond, WA, USA) and Prism 11.0.2 (GraphPad, San Diego, CA, USA). Live cell images were analyzed using the Incucyte software version 2026A (Sartorius, Göttingen, Germany), exported to Microsoft Excel as ‘red area/phase area’. Western blotting images were processed using Image Studio Lite 6.0.0.28 (LICOR bio, Lincoln, NE, USA) and confocal images were analyzed using Image J (fiji, release 2.18.0).

## 3. Results

### 3.1. Infectious HAZV with Epitope-Tagged Nucleoprotein

**Figure 1:**
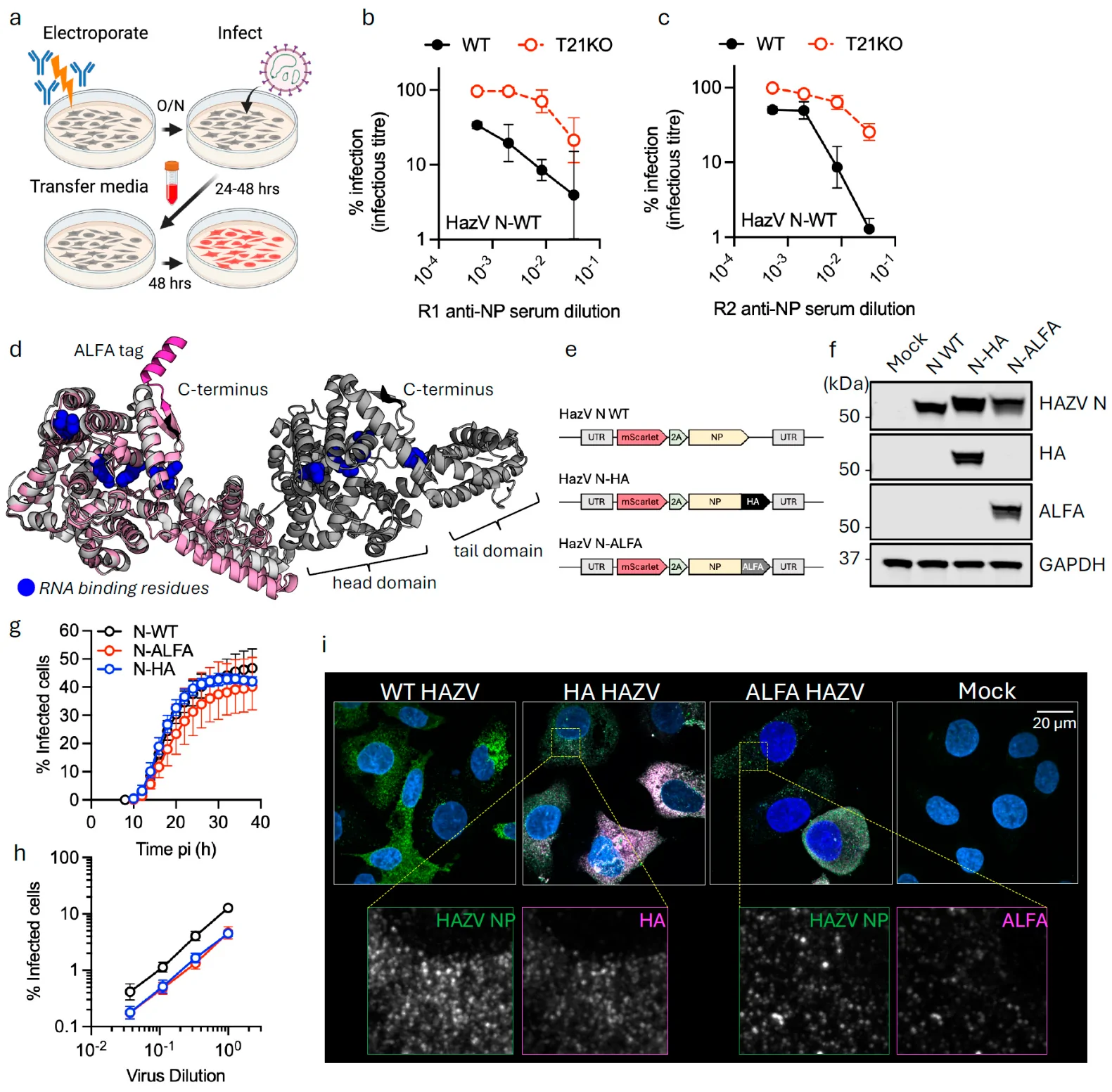
Infectious HAZV with epitope-tagged nucleoprotein (NP)**.** (**a**) Schematic of an electroporated-antibody-dependent neutralization assay (EDNA) and virus titration experiment. (**b**,**c**) Anti-NP immune sera from two rabbits were electroporated into WT and T21KO L929 cells, which were then infected with HAZV. Virus produced after 48 h was titrated onto fresh L292 cells and infection was measured by a virus-encoded fluorescence gene, normalized to 100% infection in the absence of antibody. (**d**) AF3 model of ALFA-tagged HAZV NP superposed onto the structure of head-to-tail HAZV NP oligomer from PDB 5A97. (**e**) Gene cassette showing arrangement of reporter gene and epitope-tagged NP in the HAZV S segment rescue plasmid. (**f**) Western blot using anti-NP antisera [25], anti-HA (Sigma Aldrich, #H6908) or anti-ALFA (NanoTag Biotechnologies, #N1580) antibodies. (**g**) Kinetics of virally encoded gene expression (mScarlet), as % of infected cells. (**h**) Titration of virus-containing media from (**g**) adding to fresh cells and infection measured as % of mScarlet cells. (**i**) Confocal microscopy of SW13 cells 24 h post-infection and stained with rabbit anti-NP antisera, anti-HA or anti-ALFA antibodies. Anti-NP co-localizes with anti-HA and anti-ALFA staining.

To investigate intracellular neutralization of HAZV by anti-nucleoprotein (NP) antibodies, we immunized two rabbits with recombinant NP and tested the resulting immune sera by EDNA [21]. Briefly, wildtype (WT) or TRIM21-knockout (T21KO) cells were electroporated with a range of antibody concentrations then infected the next day with a recombinant HAZV clone [24] that carries a fluorescent reporter gene for quantification. After 48 h, virus-containing media was removed and added to fresh cells to quantify the infection of produced virus (Figure 1a). Anti-NP sera from both rabbits was capable of potently blocking the production of infectious HAZV (Figure 1b,c). Neutralization was impaired in T21KO cells indicating TRIM21 potentiates HAZV inhibition by the sera. However, at high concentrations of sera, TRIM21-independent inhibition was observed, consistent with previous studies using CCHFV-immune sera [11]. Together these data confirm that antibodies against the HAZV NP can restrict HAZV replication in a TRIM21-dependent manner.

Next, to focus on the TRIM21-dependent component of HAZV neutralization and allow antiviral activity to be assessed in an epitope-independent manner, we introduced an HA or ALFA tag onto the NP C-terminus. Modeling an ALFA epitope onto an NP monomer using AlphaFold3 (pTM = 0.84) and superposing it onto the crystal structure of HAZV NP oligomer (PDB: 5A97 [26]) suggested that a C-terminal tag would not interfere with the head-to-tail packing of HAZV NP (Figure 1d). We also modified the viral genome to include an mScarlet fluorescent reporter gene followed by a 2A site and NP, either unmodified or with an HA or ALFA tag (Figure 1e). Western blotting of virus produced in SW13 cells confirmed that each construct expressed NP with the correct tag (Figure 1f). Moreover, similar levels of epitope-tagged viruses were produced as WT suggesting that the process of viral packaging was unaffected. We challenged SW13 cells with each virus to compare infectious titre and check reporter gene expression. Live imaging using an Incucyte system confirmed mScarlet reporter activity and suggested that the C-terminal epitope on NP does not substantially interfere with the efficiency of virally encoded gene expression or the production of infectious virions (Figure 1g,h). Finally, confocal microscopy of cells 24 h post-infection revealed co-localization of NP and epitope-tag staining, consistent with the expected expression behavior (Figure 1i).

### 3.2. Intracellular Antibodies Against Epitope-Tagged NP Inhibit HAZV Infection

**Figure 2:**
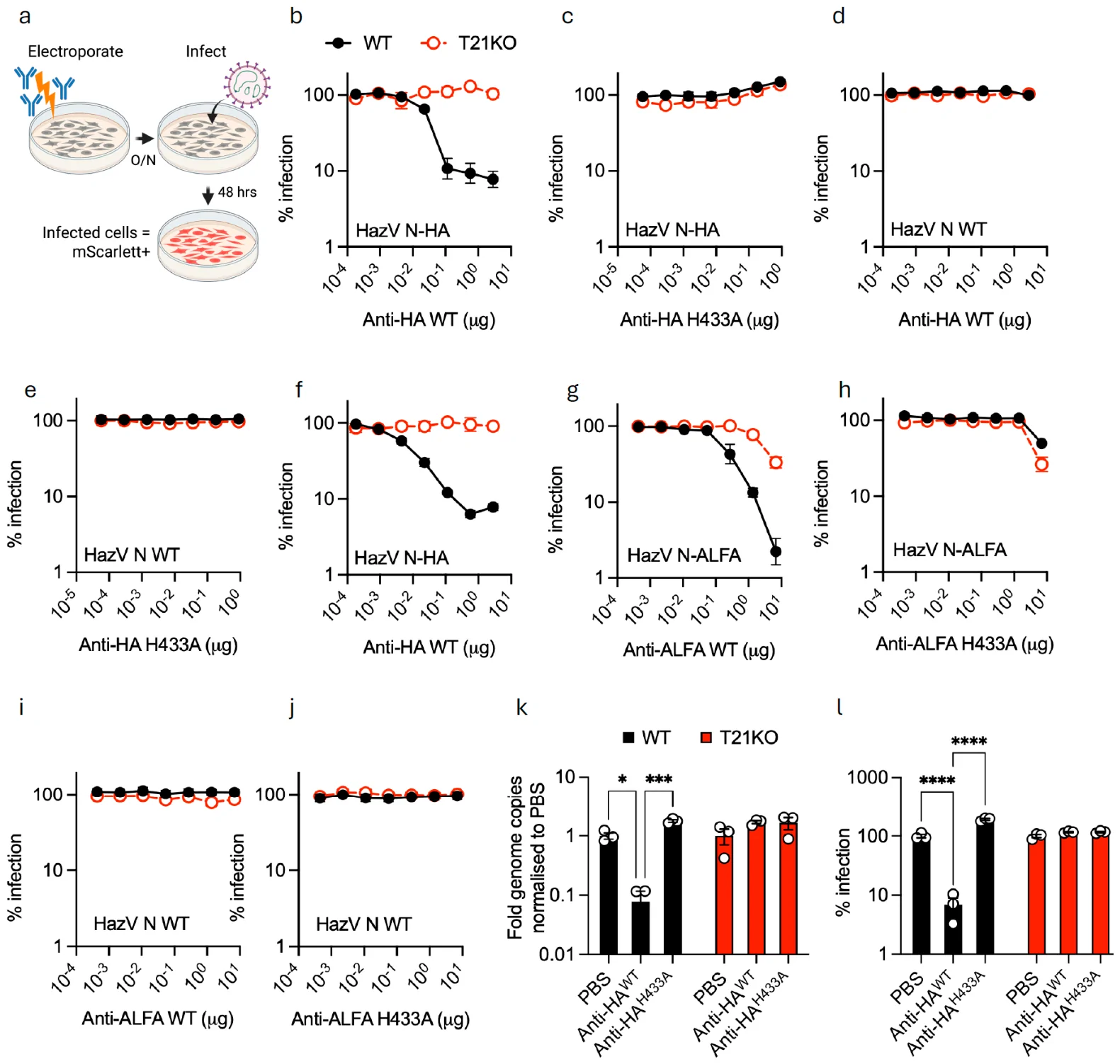
Intracellular antibodies against epitope-tagged NP inhibit HAZV infection. (**a**) Schematic of an electroporated-antibody-dependent neutralization assay (EDNA) where infection is measured as the percentage of fluorescent-positive (mScarlet) cells. (**b**–**j**) Infection of wildtype (WT) or TRIM21-knockout (T21KO) Hela cells, except (**f**) that uses L929, with HAZV that is either wildtype for NP (N-WT), HA-tagged (N-HA), or ALFA-tagged (N-ALFA). Cells were electroporated prior to infection with a titration of antibody that is either wildtype (WT) or mutated to prevent TRIM21 binding (H433A) and is specific to either HA (12CA5) or ALFA (NbALFA). The percentage of infected cells in each case was determined 48 h later using an Incucyte by calculating the % of cells that were positive for virally encoded mScarlet fluorescence. (**k**,**l**) HeLa cells were electroporated with 1 µg of antibody, infected with HAZV N-HA, media-changed and incubated for 48 h later. Virus-containing media was then removed and viral production was assessed by quantifying genomes by qPCR (**k**) and measuring their infectivity by adding to fresh cells and measuring % of infected cells by Incucyte (**l**). *, ***, and **** represent *p* < 0.05, *p* < 0.001 and *p* < 0.0001, respectively.

Next we tested whether antibodies against epitope-tagged NP can inhibit HAZV infection using an EDNA assay. Cells were electroporated with a concentration range of commercial anti-HA or anti-ALFA antibodies, then challenged with virus the following day. Infection was quantified 48 h later by measuring the percentage of fluorescent cells (Figure 2a). Electroporated humanized anti-HA antibody dose-dependently inhibited the infection of HAZV N-HA by > 1-log (Figure 2b). Importantly, neutralization was TRIM21-dependent as infection was unaffected in TRIM21 KO (T21KO) cells. TRIM21-dependence was confirmed by using anti-HA antibody with an H433A mutation that specifically ablates TRIM21 binding (Figure 2c) [27,28]. Neutralization was also lost when using WT or H433A anti-HA antibodies with WT HAZV lacking HA-tagged NP (N-WT) (Figure 2d,e). To confirm that neutralization was not cell-dependent, we repeated the EDNA experiments in mouse L929 cells. Again, we observed potent, TRIM21-dependent neutralization (Figure 2f). Next, we replaced the anti-HA with a humanized anti-ALFA nanobody-Fc antibody. We observed even more potent inhibition of infection than with anti-HA, albeit there was also some level of activity in T21KO cells (Figure 2g). Using an H433A mutant confirmed that the majority of neutralization was TRIM21-dependent with some TRIM21-independent inhibition of infection at the highest antibody concentration (Figure 2h). Infection of cells using untagged HAZV in the presence of either WT or H433A anti-ALFA antibody showed that all neutralization activity is dependent upon epitope binding (Figure 2i,j). To confirm that intracellular antibody neutralization inhibits viral replication and not just reporter gene expression, we collected viral supernatant 48 h post-infection and measured viral RNA production by qPCR and by quantifying infection in fresh cells. There were 1-log fewer virions produced from WT cells that had been infected after WT anti-HA antibody electroporation than virions produced under any other condition (T21KO cells or using anti-HA H433A antibody) and this was matched by a 1-log reduction in cell infection (Figure 2k,l). Together the data show that the presence of intracellular antibodies directed against epitope-tagged NP can inhibit HAZV infection intracellularly and this restriction requires TRIM21.

### 3.3. TRIM21 Detects Incoming NP+ Particles and Prevents Viral Transcription, Protein Expression and Genome Replication

**Figure 3:**
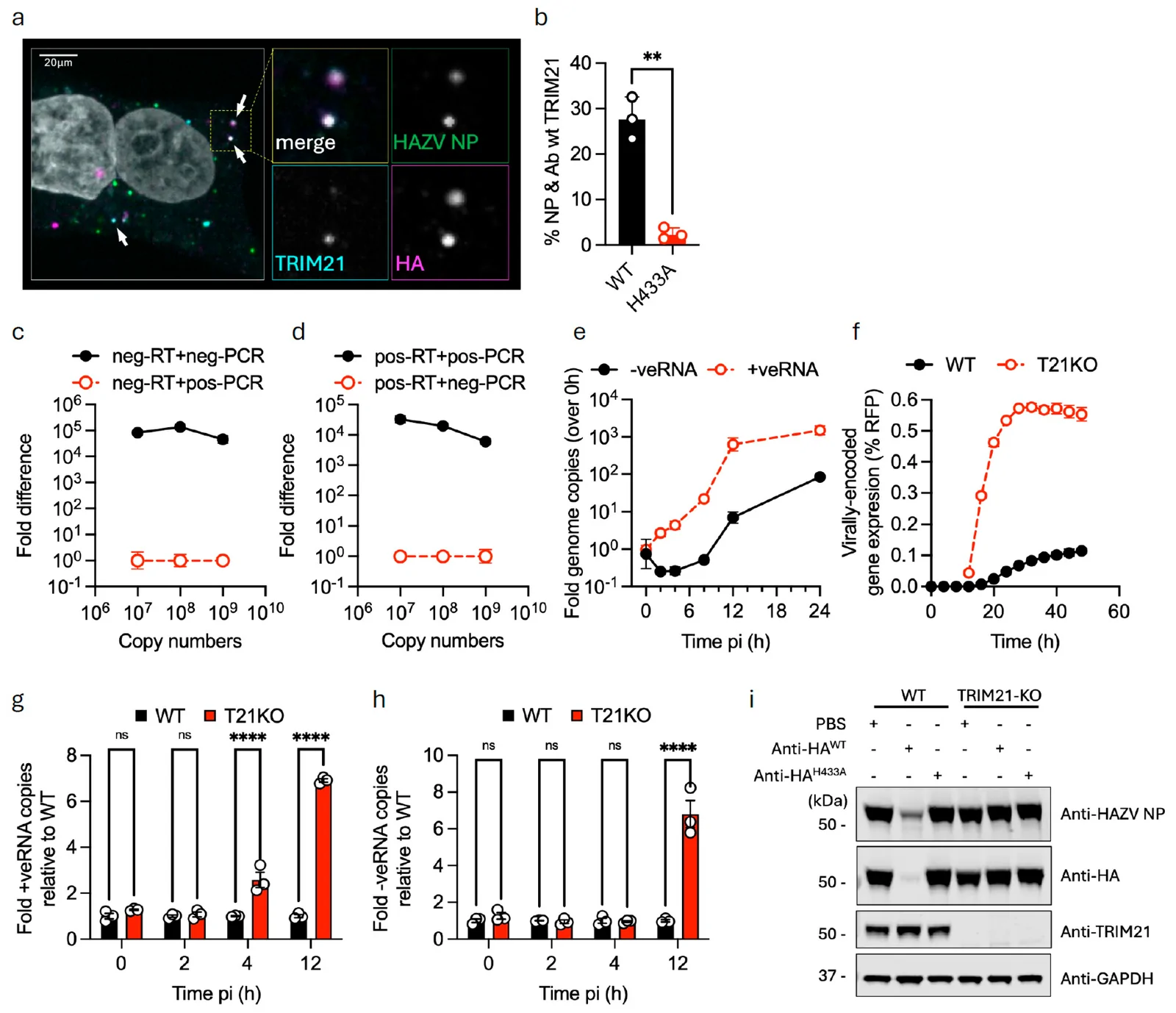
TRIM21 detects incoming NP+ particles and prevents viral transcription, protein expression and genome replication. (**a**,**b**) T21KO HEK293T cells reconstituted with FusionRed-TRIM21 and pre-electroporated with WT or H433A anti-HA antibody were infected with HAZV N-HA virus. After 2 h cells were fixed, permeabilized and stained with anti-NP antibodies and secondary against anti-HA. The resulting NP+ particles were assessed for co-localization with anti-HA antibody and FusionRed-TRIM21 (**a**) and the results quantified (**b**) (** *p* < 0.01). (**c**,**d**) Validation of strand-specific primers for detection of positive- (+ve) and negative-strand (-ve) HAZV S segment RNA. Matched and mismatched templates and qPCR primers were used to show strand-specificity for amplifying titrated amounts of strand-specific templates. RT = template, PCR = qPCR primers. (**e**) Fold-change in viral genome copies of +veRNA and -veRNA following infection of HAZV N-HA. (**f**) Infection of WT or T21KO HeLa cells after electroporation with anti-HA antibody. (**g**,**h**) As in (**e**), except fold-change in either +veRNA transcripts (**h**) or -veRNA transcripts (**i**) following infection at moi of 0.5. Significance based on one-way Anova (**** *p* < 0.0001). Data is expressed as mean and s.e.m. from at least *n* = 3 replicates. (**i**) Western blots using anti-N or anti-TRIM21 antibodies of WT or T21KO HeLa cells (TRIM21 + or -) infected with HAZV N-HA after electroporation with either WT or H433A (TRIM21 null-binding mutant) anti-HA antibody.

Previously we have shown that antibody-dependent intracellular neutralization (ADIN) allows TRIM21 to rapidly intercept incoming adenovirus virions before they can begin replicating [13,14]. Therefore, we asked whether TRIM21 can similarly detect incoming ribonucleoproteins (RNPs) from HAZV immediately after cell-viral membrane fusion. TRIM21-knockout HEK293T cells reconstituted with FusionRed TRIM21, as previously described [14], were pre-electroporated with anti-HA antibody, infected with N-HA HAZV and then fixed and stained two hours later. As shown in Figure 3a, the pre-electroporated anti-HA antibody co-localizes with NP+ particles inside the cell suggesting that incoming RNPs are detected. Importantly, TRIM21 is recruited to these HA-labeled NP+ particles (Figure 3a). Co-localization was quantified by calculating percentage of particles which were both NP+ and HA+ that were also TRIM21+ across at least five images per condition. TRIM21 recruitment is dependent upon binding to the HA antibody, as pre-electroporation with an H433A mutant anti-HA antibody prevents co-localization with NP+ particles (Figure 3b). Together with the data in Figure 2, this shows that TRIM21 neutralization of HAZV correlates with its recruitment to incoming NP+ particles.

Next we asked whether the rapid recruitment of TRIM21 to HAZV N-HA is sufficient to prevent the early steps of viral replication. HAZV is a negative-sense RNA virus and upon release of the RNPs into the cytoplasm, the viral RNA-dependent RNA polymerase must transcribe the negative-sense RNA to positive-sense mRNA to be translated by the host cell machinery. First we examined the kinetics of viral transcription and translation in the absence of neutralization. To discriminate viral transcription from later genome replication, we designed strand-specific RT and qPCR primers and showed that they were selective for either positive- or negative-strand viral RNA (Figure 3c,d), achieving up to a 5-log discrimination factor with matched template/primer combinations vs. mismatched combinations. We then used these primers to follow the kinetics of viral transcription. As shown in Figure 3e, an increase in viral transcripts (+ve RNA) was detected from 2 to 4 h post-infection and this increased until ~12 h when it plateaued, consistent with the kinetics of protein expression. Conversely, genomic (-ve RNA) transcripts did not start to increase until at least 7 h post-infection, at which point they continued to accumulate until the end of the experiment at 24 h.

We compared these results to the kinetics of virally encoded protein expression in WT or T21KO cells that had been pre-electroporated with anti-HA antibody prior to infection. Figure 3f shows that in WT cells HAZV-encoded reporter protein expression is substantially suppressed, taking longer to plateau and at much lower levels. This is restored in T21KOs, showing that suppression is TRIM21-dependent. Monitoring viral RNA levels in matched experiments revealed a significant increase in +ve RNA synthesis as early as 4 h post-infection in T21KO cells, compared to WT (Figure 3g), confirming that TRIM21-mediated ADIN blocks HAZV replication at the earliest post-entry step, consistent with the requirement for NP in viral transcription. The result of this early block is that viral replication does not proceed to genome replication, as shown by the lack of viral genome accumulation at 12 h post-infection (Figure 3h). Finally, measuring the level of HAZV NP protein by Western blot 24 h post-infection revealed that TRIM21 and intracellular antibodies block the de novo expression of NP in newly infected cells (Figure 3i). Limited N-HA was detected by both anti-NP and anti-HA antibodies with electroporation of wildtype antibody only in wildtype cells (lane 2) compared to all other conditions. This likely corresponds to minimal transcription and translation of de novo NP with ADIN. Our data supports a model in which the potent inhibition of HAZV by ADIN is a result of rapid detection of incoming RNPs by cytoplasmic antibodies and TRIM21. This subsequently blocks early viral transcription and prevents the expression of viral proteins, including de novo NP, necessary to carry out the viral life cycle.

### 3.4. TRIM21 Inhibits HAZV Using Two Different Mechanisms

**Figure 4:**
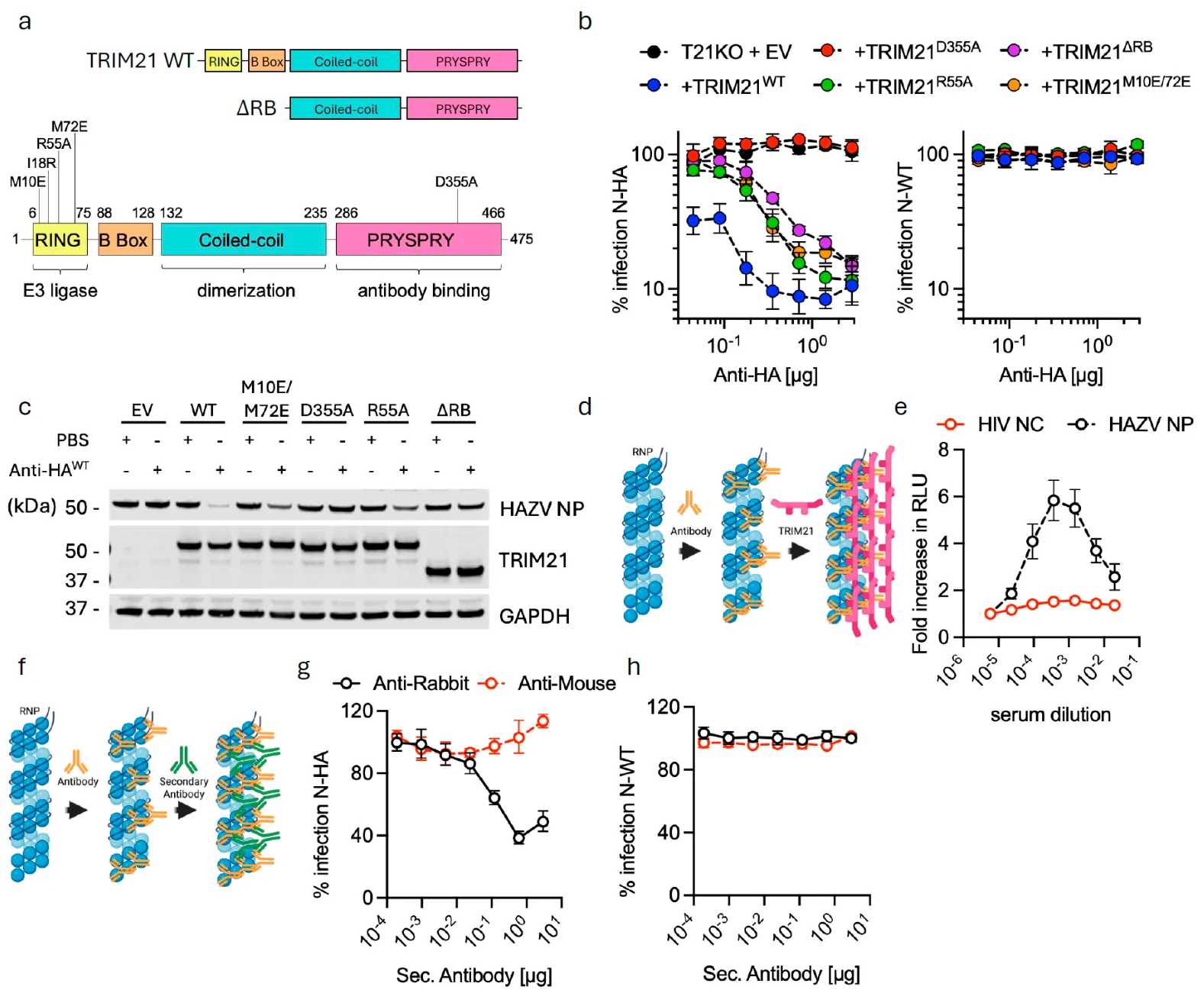
TRIM21 inhibits HAZV using two different mechanisms. (**a**) Schematic of TRIM21 protein showing domains and mutations. (**b**) T21KO HeLa cells reconstituted with indicated TRIM21 constructs and used in an EDNA assay with HAZV N-HA (left-hand side) or N-WT (right-hand side). Percentage of infected cells were measured at 48 h, normalized to no anti-HA antibody condition. (**c**) Cells infected after electroporation of 0.1775 ug of anti-HA antibody and blotted 48 h post-infection for HAZV NP protein and TRIM21. (**d**) Schematic illustrating that antibody-dependent recruitment of TRIM21 to an RNP could result in potential cross-linking of the particle in multiple TRIM21 molecules. (**e**) Recombinant HIV nucleocapsid (NC) or HAZV rNP was added to SmBiT-TRIM21 and LgBiT-TRIM21 recombinant proteins in the presence of increasing concentrations of sheep anti-NP antisera. After the addition of luciferase substrate the change in relative light units (RLUs) was measured compared to background. (**f**) Schematic illustrating that a secondary antibody could cross-link antibody-bound RNP similarly to TRIM21. (**g**,**h**) Infection of HeLa cells with HAZV N-HA (**g**) or N-WT (**h**) 12 h after their electroporation with 0.12 µg of anti-HA rabbit antibody and titrated amounts of either anti-rabbit or anti-mouse secondary antibody. The percentage of infected cells was then measured by fluorescence, normalized to no antibody controls, 48 h post-infection.

TRIM21-mediated neutralization is dependent upon both TRIM21s’ antibody-binding and E3 ubiquitin ligase activity [13]. The latter requires clustering of multiple TRIM21 molecules, which allows the TRIM21 RING domain to dimerise and begin catalyzing ubiquitination, ultimately leading to substrate degradation [16]. To investigate the mechanism of TRIM21-mediated neutralization we reconstituted T21KO HeLa cells with either wildtype TRIM21 or TRIM21 with various mutations or domain deletions (Figure 4a) and challenged these cells with HAZV N-WT or N-HA (Figure 4b). As expected, infection of N-WT was unaffected in any cell line at any anti-HA concentration. In contrast, cells reconstituted with WT TRIM21 and challenged with N-HA showed potent dose-dependent neutralization (Figure 4b). Consistent with a role for direct antibody-binding, the D355A mutation in the PRYSPRY that prevents interaction with the ‘HNHY’ motif in IgG Fc abolished neutralization and phenocopied TRIM21 KO. Unexpectedly, mutation of RING domain residues responsible for catalysis (R55A) or RING dimerization (M10E/M72E) had an intermediate phenotype in which substantial neutralization was lost at all but the highest antibody concentrations (Figure 4b,c). The same was true for deletion of the RING and B Box domains (TRIM21^ΔRB^), which does not prevent high-affinity binding to antibody or clustering of TRIM21 but completely abolishes ubiquitination activity [15].

The above phenomenon was reminiscent to us of the antiviral activity of TRIM21s’ close homolog TRIM5, which restricts HIV-1 replication using two different mechanisms [29,30,31]. First, TRIM5 forms a lattice or ‘cage’ around the incoming retroviral capsid that physically blocks infection by preventing nuclear entry [32,33]. Second, the TRIM5 RING domains catalyze ubiquitination and direct the capsid for degradation, resulting in the loss of viral DNA [34]. Importantly, only the second mechanism is RING and ubiquitination-dependent: RING mutations prevent degradation and restore reverse transcription but do not rescue infection. We hypothesized that the RING-independent component of TRIM21-mediated HAZV neutralization could be the result of a similar caging effect, in which cross-linking of RNPs by TRIM21 interferes with NP function (Figure 4d). To test this we performed a split-luciferase assay in which two versions of full-length TRIM21 protein, one fused to LgBiT luciferase and one to SmBiT luciferase, were mixed with either HAZV NP or HIV nucleocapsid (NC) under increasing concentrations of anti-NP serum (Figure 4e). Only if multiple TRIM21 molecules are clustered on an antibody-coated target will a luminescent signal be generated, as previously shown for TRIM21 recruitment to adenovirus capsid [14]. We observed a dose-dependent increase in luciferase signal when using HAZV NP, followed by a decrease at higher antibody concentrations. This is consistent with the recruitment of multiple TRIM21 molecules to antibody-bound NP until antibodies are in excess over NP, at which point 1:1 TRIM21:Ab complexes are formed and active luciferase is no longer generated. No luciferase signal was observed with HIV NC. This data is consistent with a similar caging model of restriction as in TRIM5.

To test this hypothesis further we asked whether a similar effect could be achieved if we replaced TRIM21 with a secondary antibody that could also cross-link NP through the primary antibody (Figure 4f). We electroporated TRIM21 KO cells with rabbit anti-HA antibody and titrated amounts of either anti-rabbit or anti-mouse secondary, then infected them with HAZV N-WT or N-HA. HAZV N-WT was unaffected, as was N-HA when anti-mouse secondary was used. However, anti-rabbit secondary led to a modest but dose-dependent decrease in N-HA infection, consistent with physical caging acting to inhibit HAZV replication (Figure 4g). The smaller phenotype is likely because secondary antibodies cannot interact with each other, whereas TRIM21 molecules can form higher-order structures via RING dimerization, greatly potentiating the caging effect.

### 3.5. ADIN Blocks HAZV Replication Post-Infection

**Figure 5:**
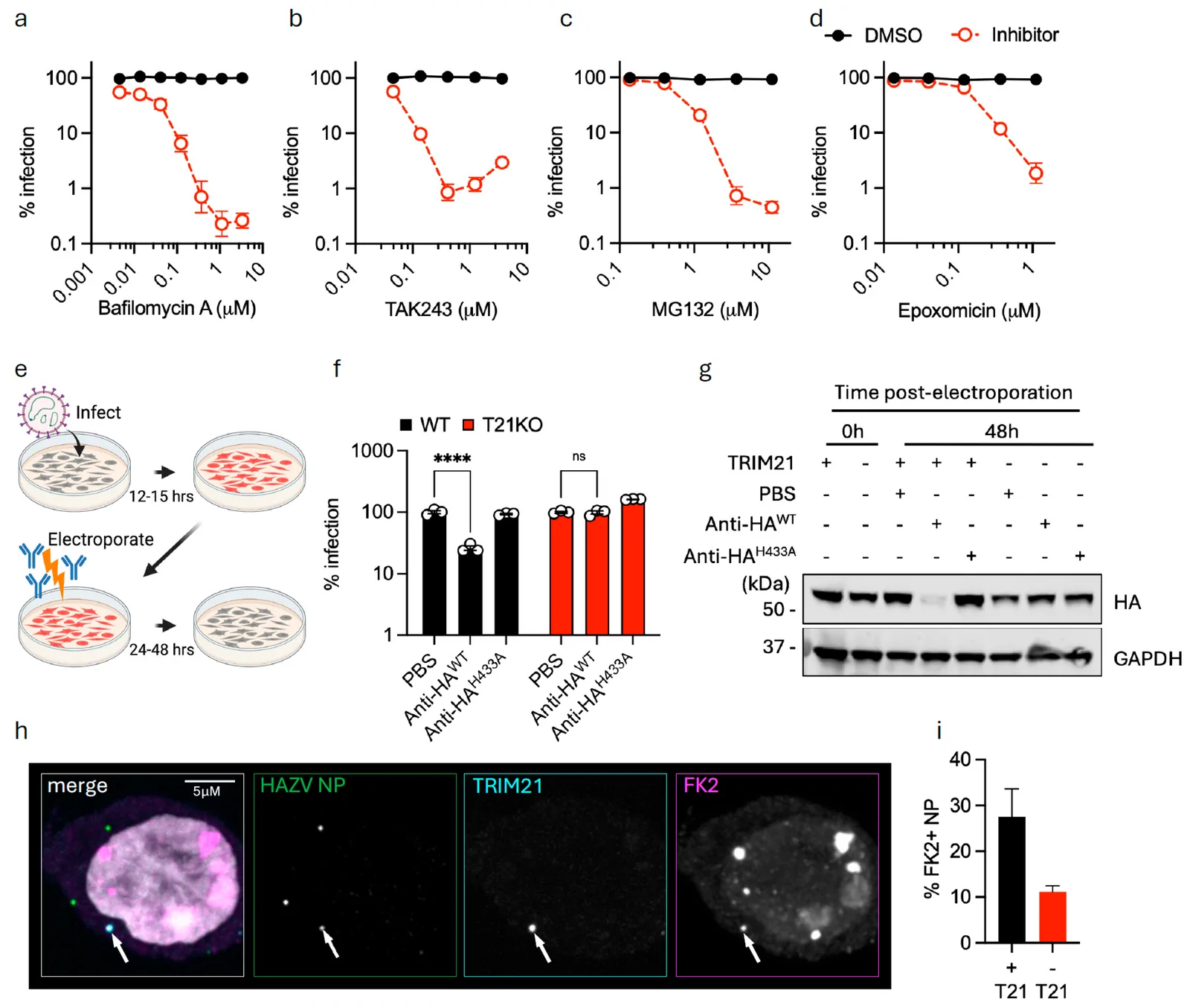
ADIN blocks HAZV replication post-infection. (**a**–**d**) Vero cells were infected with HAZV N-WT in the presence of the indicated inhibitors and infection measured 12 h later. (**e**) Schematic showing variation in EDNA assay in which cells are infected and then electroporated with antibodies after 12–15 h. Infection is then measured 24–48 h later. (**f**) Relative infection levels for experiment carried out as in (**e**) in WT or T21KO cells challenged with HAZV N-HA and electroporated with wildtype or H433A anti-HA antibody (**** *p* < 0.0001). (**g**) As in (**f**), except cells were Western blotted for NP using anti-HA antibody. (**h**) HEK293T cells expressing FusionRed-TRIM21 were electroporated with human anti-HA antibody then infected with HAZV N-HA. The 2 h post-infection cells were fixed and stained with mouse anti-FK2 (to detect ubiquitin) and rabbit anti-HAZV NP. (**i**) Quantification (percentage) of NP puncta that are FK2-positive and either TRIM21-positive or -negative.

While the above data suggest that TRIM21 binding may reduce HAZV infection by physically caging RNPs, there is still a component of restriction that is RING-dependent (Figure 4b). This RING-dependent phenotype suggests infection could be inhibited by ubiquitination-mediated degradation of NP. Previously we have used inhibitors of either the autophagy pathway or the ubiquitin–proteasome system (UPS) to investigate substrate degradation by TRIM21 [14]. However, when testing inhibitors of these pathways we observed that addition of E1 inhibitor (TAK243) or proteasome inhibitors (MG132 or epoxomicin) led to a reduction in HAZV infection levels in the absence of antibody, analogous to bafilomycin A used here as a positive control (Figure 5a–d). This suggested that HAZV replication is dependent on ubiquitination and the proteasome. We therefore took a different approach and asked whether TRIM21 could reverse or limit infection after replication had begun. We tested this by challenging WT or T21KO cells with HAZV N-WT or N-HA, then media-changing after 12 h to remove virus, followed by electroporation with anti-HA antibody (Figure 5e). Figure 5f shows that in WT cells electroporated with WT anti-HA antibody there was significantly lower infection levels as measured 24 h later. To link this effect to the degradation of NP protein already made by the virus, we repeated the experiment electroporating cells with anti-HA antibodies 15 h post-infection and compared NP levels by Western blot pre- and post-electroporation. Substantial NP was present in cells after 15 h (0 h post-electroporation) and these levels persisted 48 h later in all cells except those containing TRIM21 and anti-HA antibodies (Figure 5g). This data suggests that TRIM21 is capable of halting replication post-infection by degrading pre-existing NP protein. Finally, to test whether TRIM21 detection of HAZV RNP is associated with particle ubiquitination, we fixed anti-HA-electroporated cells 2 h post-infection and blotted with the anti-ubiquitin antibody FK2. Figure 5h,i show that there is an increase in ubiquitin-positive NP puncta in the presence of TRIM21, consistent with TRIM21-catalyzed NP ubiquitination.

## 4. Discussion

Neutralizing antibodies (nAbs) are overwhelmingly used as the principal correlate of protection for assessing both natural and vaccine-induced immunity. Despite this, non-neutralizing antibodies (nnAbs) against internal antigens (e.g., nucleoprotein (NP)) are often produced at levels that match or exceed nAbs [35] and can arise much earlier during infection [36,37]. This could be due to the relative abundance of NP compared to other viral proteins. Also, viruses utilize mechanisms such as conformational diversity and glycan shields to reduce the antigenicity of specific proteins like envelope glycoproteins. Indeed, LCMV can be cleared in mice within two weeks [38], before a neutralizing titre is typically observed [39]. In humans infected with CCHFV, neutralizing antibodies often arise weeks after resolution of acute disease [40]. Non-neutralizing antibodies are protective in vivo against a broad range of viruses, including SARS-CoV-2 [41], human immunodeficiency virus (HIV) [42], human cytomegalovirus (HCMV) [43], bunyaviruses [1,6,8] and influenza virus [44,45], but the mechanism in many cases is unclear. Understanding the role of nnAbs and their mode of action is all the more pertinent given that vaccines against internal antigens like NP can be highly protective [5,20,44] and, because NP is typically more conserved than receptor glycoproteins, may be more widely effective [46] and resistant to evasion [47].

Here we have used an in vitro neutralization assay, EDNA, to investigate the ability of nnAbs to block the infection of HAZV, a model virus for CCHFV which requires maximum biocontainment laboratories. The data show that antibodies against tagged nucleoprotein can recognize the incoming viral nucleoprotein inside cells, resulting in the rapid recruitment of the cytosolic antibody receptor TRIM21. Upon recruitment, TRIM21 inhibits viral transcription and subsequent protein expression—reducing both primary infection and the production of new infectious virions. Investigating the mechanism of this protection revealed that TRIM21 blocks infection by two mechanisms, one RING-independent and one RING-dependent. The former mechanism we hypothesize leads to ‘caging’ of viral nucleoproteins, thereby interfering with essential functions such as interaction with host proteins to promote viral replication [48,49] or preventing the viral RNA-dependent RNA polymerase from processing through the NP-coated viral genome [50,51]. A similar RING-independent caging has been observed for TRIM5, whose binding to retroviral capsids similarly blocks infection [32,52]. For TRIM5, a second RING-dependent mechanism leads to degradation of the capsid and removal of newly transcribed viral DNA [30]. The partial loss of HAZV neutralization in cells expressing RING mutants suggests a similar degradation mechanism may be involved in the case of TRIM21-recognition of HAZV NP. However, whilst we were able to confirm TRIM21-mediated ubiquitination of NP complexes inside HAZV infected cells, we were unable to directly show proteasome-dependent NP degradation due to the reduction in infection caused by proteasome inhibition.

Our results illustrate that TRIM21-dependendent neutralization can be studied in isolation using viruses expressing epitope-tagged nucleoprotein. However, a polyclonal response will target diverse epitopes within NP itself. The fact that significant neutralization was observed when using rabbit polyclonal anti-NP antibodies in TRIM21-knockout cells highlights that a natural immune response will contain antibodies with the potential to neutralize using a variety of mechanisms that may include directly competing with functional interfaces on NP. Whilst our data show that nnAbs can be neutralizing once inside the cell they do not explain how nnAbs reach the inside of cells during natural infection. In this regard it is noteworthy that while viral nucleoproteins typically contain neither transmembrane domains nor secretory signals, they are often found on the plasma membrane of infected cells [53,54,55,56,57] or secreted [58,59]. Moreover, extracellular NP can be taken up and internalized by uninfected cells [55,56,59,60,61]. Multiple mechanisms have been postulated to explain this behavior, including unconventional protein secretion [62], extracellular vesicles [63,64] and direct protein translocation across cellular membranes [65]. It is tempting to speculate that anti-NP antibodies could hitch a ride into cells as a complex with free NP. Certainly the cellular tropism of viruses like HAZV and CCHFV includes highly phagocytic cells like macrophages, dendritic cells and hepatocytes that express both scavenging receptors and FcγRs. Further work is needed to understand where in vivo anti-NP antibodies encounter NP protein, how they are utilized inside cells by TRIM21 to prevent infection, and how they contribute to vaccine protection.

## Data Availability

The original contributions presented in this study are included in the article/Supplementary Material. Further inquiries can be directed to the corresponding author.

## Supplementary Materials

The following supporting information can be downloaded at https://www.mdpi.com/article/10.3390/v18090974/s1, Table S1: List of primers and oligo standards used; Table S2: List of plasmids used; Table S3: List of antibodies used.

## References

[1] Hawman D.W., Ahlen G., Appelberg K.S., Meade-White K., Hanley P.W., Scott D., Monteil V., Devignot S., Okumura A., Weber F. (2021). A DNA-based vaccine protects against Crimean-Congo haemorrhagic fever virus disease in a Cynomolgus macaque model. Nat. Microbiol..

[2] Hawman D.W., Meade-White K., Leventhal S., Appelberg S., Ahlén G., Nikouyan N., Clancy C., Smith B., Hanley P., Lovaglio J. (2023). Accelerated DNA vaccine regimen provides protection against Crimean-Congo hemorrhagic fever virus challenge in a macaque model. Mol. Ther..

[3] Rao J., Li J., Jiang T., Guo W., Zhang X., Zhang Z., Su J., Lu M., Hu X., Liu X. (2025). A single-dose mRNA vaccine protects mice from lethal Crimean-Congo hemorrhagic fever virus infection. npj Vaccines.

[4] Tipih T., Leventhal S.S., Meade-White K., Lewis M., Bushmaker T., Shaia C., Marzi A., Feldmann H., Hawman D.W. (2025). Single dose VSV-based vaccine protects mice against lethal heterologous Crimean-Congo hemorrhagic fever virus challenge. npj Vaccines.

[5] Leventhal S.S., Meade-White K., Rao D., Haddock E., Leung J., Scott D., Archer J., Randall S., Erasmus J.H., Feldmann H. (2022). Replicating RNA vaccination elicits an unexpected immune response that efficiently protects mice against lethal Crimean-Congo hemorrhagic fever virus challenge. eBioMedicine.

[6] Sorvillo T.E., Karaaslan E., Scholte F.E.M., Welch S.R., Coleman-McCray J.D., Genzer S.C., Ritter J.M., Hayes H.M., Jain S., Pegan S.D. (2024). Replicon particle vaccination induces non-neutralizing anti-nucleoprotein antibody-mediated control of Crimean-Congo hemorrhagic fever virus. npj Vaccines.

[7] Appelberg S., John L., Pardi N., Végvári Á., Bereczky S., Ahlén G., Monteil V., Abdurahman S., Mikaeloff F., Beattie M. (2021). Nucleoside-modified mRNA vaccines protect IFNAR(-/-) mice against Crimean Congo hemorrhagic fever virus infection. J. Virol..

[8] Zivcec M., Safronetz D., Scott D.P., Robertson S., Feldmann H. (2018). Nucleocapsid protein-based vaccine provides protection in mice against lethal Crimean-Congo hemorrhagic fever virus challenge. PLoS Neglected Trop. Dis..

[9] Garrison A.R., Moresco V., Zeng X., Cline C.R., Ward M.D., Ricks K.M., Olschner S.P., Cazares L.H., Karaaslan E., Fitzpatrick C.J. (2024). Nucleocapsid protein-specific monoclonal antibodies protect mice against Crimean-Congo hemorrhagic fever virus. Nat. Commun..

[10] Hawman D.W., Leventhal S., Meade-White K., Graham W., Gaffney K., Khandhar A., Murray J., Prado-Smith J., Shaia C., Saturday G. (2025). A replicating RNA vaccine confers protection against Crimean-Congo hemorrhagic fever in cynomolgus macaques. eBioMedicine.

[11] Leventhal S.S., Bisom T., Clift D., Rao D., Meade-White K., Shaia C., Murray J., Mihalakakos E.A., Hinkley T., Reynolds S.J. (2024). Antibodies targeting the Crimean-Congo Hemorrhagic Fever Virus nucleoprotein protect via TRIM21. Nat. Commun..

[12] James L.C., Keeble A.H., Khan Z., Rhodes D.A., Trowsdale J. (2007). Structural basis for PRYSPRY-mediated tripartite motif (TRIM) protein function. Proc. Natl. Acad. Sci. USA.

[13] Mallery D.L., McEwan W.A., Bidgood S.R., Towers G.J., Johnson C.M., James L.C. (2010). Antibodies mediate intracellular immunity through tripartite motif-containing 21 (TRIM21). Proc. Natl. Acad. Sci. USA.

[14] Rhinesmith T., Albecka A., Vaysburd M., Puri C., Luptak J., Boulanger J., Nguyen Le Q.M., Gratian M.J., O’Connell K., Few L. (2026). TRIM21 induces selective autophagy of viruses and bacteria. Mol. Cell.

[15] Dickson C., Fletcher A.J., Vaysburd M., Yang J.C., Mallery D.L., Zeng J., Johnson C.M., McLaughlin S.H., Skehel M., Maslen S. (2018). Intracellular antibody signalling is regulated by phosphorylation of the Fc receptor TRIM21. eLife.

[16] Zeng J., Santos A.F., Mukadam A.S., Osswald M., Jacques D.A., Dickson C.F., McLaughlin S.H., Johnson C.M., Kiss L., Luptak J. (2021). Target-induced clustering activates Trim-Away of pathogens and proteins. Nat. Struct. Mol. Biol..

[17] Clift D., McEwan W.A., Labzin L.I., Konieczny V., Mogessie B., James L.C., Schuh M. (2017). A Method for the Acute and Rapid Degradation of Endogenous Proteins. Cell.

[18] McEwan W.A., Falcon B., Vaysburd M., Clift D., Oblak A.L., Ghetti B., Goedert M., James L.C. (2017). Cytosolic Fc receptor TRIM21 inhibits seeded tau aggregation. Proc. Natl. Acad. Sci. USA.

[19] Caddy S.L., Vaysburd M., Papa G., Wing M., O’Connell K., Stoycheva D., Foss S., Terje Andersen J., Oxenius A., James L.C. (2020). Viral nucleoprotein antibodies activate TRIM21 and induce T cell immunity. EMBO J..

[20] Stamper A., Bisom T., Meade-White K., Lewis M., Leventhal S., Clancy C., Hinkley T., Erasmus J., Feldmann H., Hawman D.W. (2025). A replicating RNA vaccine protects against severe fever with thrombocytopenia syndrome virus infection in mice. npj Vaccines.

[21] Albecka A., Clift D., Vaysburd M., Rhinesmith T., Caddy S.L., Favara D.M., Baxendale H.E., James L.C. (2021). A functional assay for serum detection of antibodies against SARS-CoV-2 nucleoprotein. EMBO J..

[22] Kalkan-Yazici M., Karaaslan E., Cetin N.S., Hasanoglu S., Guney F., Zeybek U., Doymaz M.Z. (2021). Cross-Reactive anti-Nucleocapsid Protein Immunity against Crimean-Congo Hemorrhagic Fever Virus and Hazara Virus in Multiple Species. J. Virol..

[23] Bottermann M., Foss S., Caddy S.L., Clift D., van Tienen L.M., Vaysburd M., Cruickshank J., O’Connell K., Clark J., Mayes K. (2019). Complement C4 Prevents Viral Infection through Capsid Inactivation. Cell Host Microbe.

[24] Fuller J., Surtees R.A., Slack G.S., Mankouri J., Hewson R., Barr J.N. (2019). Rescue of Infectious Recombinant Hazara Nairovirus from cDNA Reveals the Nucleocapsid Protein DQVD Caspase Cleavage Motif Performs an Essential Role other than Cleavage. J. Virol..

[25] Fuller J., Álvarez-Rodríguez B., Todd E.J.A.A., Mankouri J., Hewson R., Barr J.N. (2020). Hazara Nairovirus Requires COPI Components in both Arf1-Dependent and Arf1-Independent Stages of Its Replication Cycle. J. Virol..

[26] Surtees R., Ariza A., Punch E.K., Trinh C.H., Dowall S.D., Hewson R., Hiscox J.A., Barr J.N., Edwards T.A. (2015). The crystal structure of the Hazara virus nucleocapsid protein. BMC Struct. Biol..

[27] Foss S., Watkinson R.E., Grevys A., McAdam M.B., Bern M., Hoydahl L.S., Dalhus B., Michaelsen T.E., Sandlie I., James L.C. (2016). TRIM21 Immune Signaling Is More Sensitive to Antibody Affinity Than Its Neutralization Activity. J. Immunol..

[28] McEwan W.A., Hauler F., Williams C.R., Bidgood S.R., Mallery D.L., Crowther R.A., James L.C. (2012). Regulation of virus neutralization and the persistent fraction by TRIM21. J. Virol..

[29] Fletcher A.J., Vaysburd M., Maslen S., Zeng J., Skehel J.M., Towers G.J., James L.C. (2018). Trivalent RING Assembly on Retroviral Capsids Activates TRIM5 Ubiquitination and Innate Immune Signaling. Cell Host Microbe.

[30] Stremlau M., Owens C.M., Perron M.J., Kiessling M., Autissier P., Sodroski J. (2004). The cytoplasmic body component TRIM5alpha restricts HIV-1 infection in Old World monkeys. Nature.

[31] Stremlau M., Perron M., Lee M., Li Y., Song B., Javanbakht H., Diaz-Griffero F., Anderson D.J., Sundquist W.I., Sodroski J. (2006). Specific recognition and accelerated uncoating of retroviral capsids by the TRIM5alpha restriction factor. Proc. Natl. Acad. Sci. USA.

[32] Li Y.L., Chandrasekaran V., Carter S.D., Woodward C.L., Christensen D.E., Dryden K.A., Pornillos O., Yeager M., Ganser-Pornillos B.K., Jensen G.J. (2016). Primate TRIM5 proteins form hexagonal nets on HIV-1 capsids. eLife.

[33] Skorupka K.A., Roganowicz M.D., Christensen D.E., Wan Y., Pornillos O., Ganser-Pornillos B.K. (2019). Hierarchical assembly governs TRIM5alpha recognition of HIV-1 and retroviral capsids. Sci. Adv..

[34] Campbell E.M., Weingart J., Sette P., Opp S., Sastri J., O’Connor S.K., Talley S., Diaz-Griffero F., Hirsch V., Bouamr F. (2016). TRIM5alpha-Mediated Ubiquitin Chain Conjugation Is Required for Inhibition of HIV-1 Reverse Transcription and Capsid Destabilization. J. Virol..

[35] Rydyznski Moderbacher C., Ramirez S.I., Dan J.M., Grifoni A., Hastie K.M., Weiskopf D., Belanger S., Abbott R.K., Kim C., Choi J. (2020). Antigen-Specific Adaptive Immunity to SARS-CoV-2 in Acute COVID-19 and Associations with Age and Disease Severity. Cell.

[36] Battegay M., Moskophidis D., Waldner H., Brundler M.A., Fung-Leung W.P., Mak T.W., Hengartner H., Zinkernagel R.M. (1993). Impairment and delay of neutralizing antiviral antibody responses by virus-specific cytotoxic T cells. J. Immunol..

[37] Eschli B., Zellweger R.M., Wepf A., Lang K.S., Quirin K., Weber J., Zinkernagel R.M., Hengartner H. (2007). Early antibodies specific for the neutralizing epitope on the receptor binding subunit of the lymphocytic choriomeningitis virus glycoprotein fail to neutralize the virus. J. Virol..

[38] Moskophidis D., Lechner F., Pircher H., Zinkernagel R.M. (1993). Virus persistence in acutely infected immunocompetent mice by exhaustion of antiviral cytotoxic effector T cells. Nature.

[39] Fallet B., Hao Y., Florova M., Cornille K., de Los Aires A.V., Girelli Zubani G., Ertuna Y.I., Greiff V., Menzel U., Hammad K. (2020). Chronic Viral Infection Promotes Efficient Germinal Center B Cell Responses. Cell Rep..

[40] Shepherd A.J., Swanepoel R., Leman P.A. (1989). Antibody response in Crimean-Congo hemorrhagic fever. Rev. Infect. Dis..

[41] Dangi T., Sanchez S., Class J., Richner M., Visvabharathy L., Chung Y.R., Bentley K., Stanton R.J., Koralnik I.J., Richner J.M. (2022). Improved control of SARS-CoV-2 by treatment with a nucleocapsid-specific monoclonal antibody. J. Clin. Investig..

[42] Mayr L.M., Su B., Moog C. (2017). Non-Neutralizing Antibodies Directed against HIV and Their Functions. Front. Immunol..

[43] Bootz A., Karbach A., Spindler J., Kropff B., Reuter N., Sticht H., Winkler T.H., Britt W.J., Mach M. (2017). Protective capacity of neutralizing and non-neutralizing antibodies against glycoprotein B of cytomegalovirus. PLoS Pathog..

[44] Carragher D.M., Kaminski D.A., Moquin A., Hartson L., Randall T.D. (2008). A novel role for non-neutralizing antibodies against nucleoprotein in facilitating resistance to influenza virus. J. Immunol..

[45] Sambhara S., Kurichh A., Miranda R., Tumpey T., Rowe T., Renshaw M., Arpino R., Tamane A., Kandil A., James O. (2001). Heterosubtypic immunity against human influenza A viruses, including recently emerged avian H5 and H9 viruses, induced by FLU-ISCOM vaccine in mice requires both cytotoxic T-lymphocyte and macrophage function. Cell. Immunol..

[46] Fujimoto Y., Tomioka Y., Takakuwa H., Uechi G.I., Yabuta T., Ozaki K., Suyama H., Yamamoto S., Morimatsu M., Mai L.Q. (2016). Cross-protective potential of anti-nucleoprotein human monoclonal antibodies against lethal influenza A virus infection. J. Gen. Virol..

[47] Shu L.L., Bean W.J., Webster R.G. (1993). Analysis of the evolution and variation of the human influenza A virus nucleoprotein gene from 1933 to 1990. J. Virol..

[48] Ali S., Ren S., Agsaoa A., Mir S., Mir M.A. (2025). The nucleocapsid protein of Crimean–Congo hemorrhagic fever virus interacts with eIF4A to promote the translation of viral mRNA in cells. J. Biol. Chem..

[49] Andersson I., Simon M., Lundkvist A., Nilsson M., Holmström A., Elgh F., Mirazimi A. (2004). Role of actin filaments in targeting of Crimean Congo hemorrhagic fever virus nucleocapsid protein to perinuclear regions of mammalian cells. J. Med. Virol..

[50] Green Todd J., Cox R., Tsao J., Rowse M., Qiu S., Luo M. (2014). Common Mechanism for RNA Encapsidation by Negative-Strand RNA Viruses. J. Virol..

[51] Sabsay K.R., Te Velthuis A.J.W. (2023). Negative and ambisense RNA virus ribonucleocapsids: More than protective armor. Microbiol. Mol. Biol. Rev..

[52] Wagner J.M., Roganowicz M.D., Skorupka K., Alam S.L., Christensen D., Doss G., Wan Y., Frank G.A., Ganser-Pornillos B.K., Sundquist W.I. (2016). Mechanism of B-box 2 domain-mediated higher-order assembly of the retroviral restriction factor TRIM5alpha. eLife.

[53] Yewdell J.W., Frank E., Gerhard W. (1981). Expression of influenza A virus internal antigens on the surface of infected P815 cells. J. Immunol..

[54] Yewdell J.W., Bennink J.R., Mackett M., Lefrancois L., Lyles D.S., Moss B. (1986). Recognition of cloned vesicular stomatitis virus internal and external gene products by cytotoxic T lymphocytes. J. Exp. Med..

[55] Marie J.C., Saltel F., Escola J.M., Jurdic P., Wild T.F., Horvat B. (2004). Cell surface delivery of the measles virus nucleoprotein: A viral strategy to induce immunosuppression. J. Virol..

[56] Lopez-Munoz A.D., Kosik I., Holly J., Yewdell J.W. (2022). Cell surface SARS-CoV-2 nucleocapsid protein modulates innate and adaptive immunity. Sci. Adv..

[57] Cespedes P.F., Bueno S.M., Ramirez B.A., Gomez R.S., Riquelme S.A., Palavecino C.E., Mackern-Oberti J.P., Mora J.E., Depoil D., Sacristan C. (2014). Surface expression of the hRSV nucleoprotein impairs immunological synapse formation with T cells. Proc. Natl. Acad. Sci. USA.

[58] Lopez-Munoz A.D., Yewdell J.W. (2024). Cell surface RNA virus nucleocapsid proteins: A viral strategy for immunosuppression?. npj Viruses.

[59] Albornoz A., Salazar-Quiroz N., Bignon E.A., Muena N.A., Gonzalez D., Espinoza J., Fuenzalida M.J., López-Lastra M., Ulrich R.G., Tischler N.D. (2025). Cell surface localization and intercellular transfer of the hantavirus nucleocapsid protein via a viral assembly-independent mechanism. bioRxiv.

[60] Laine D., Trescol-Biemont M.C., Longhi S., Libeau G., Marie J.C., Vidalain P.O., Azocar O., Diallo A., Canard B., Rabourdin-Combe C. (2003). Measles virus (MV) nucleoprotein binds to a novel cell surface receptor distinct from FcgammaRII via its C-terminal domain: Role in MV-induced immunosuppression. J. Virol..

[61] Fahoum J., Billan M., Varga J.K., Padawer D., Britan-Rosich Y., Elgrably-Weiss M., Basu P., Stolovich-Rain M., Baraz L., Cohen-Kfir E. (2025). Transfer of SARS-CoV-2 nucleocapsid protein to uninfected epithelial cells induces antibody-mediated complement deposition. Cell Rep..

[62] Sparn C., Meyer A., Saleppico R., Nickel W. (2022). Unconventional secretion mediated by direct protein self-translocation across the plasma membranes of mammalian cells. Trends Biochem. Sci..

[63] Zabrodskaya Y., Plotnikova M., Gavrilova N., Lozhkov A., Klotchenko S., Kiselev A., Burdakov V., Ramsay E., Purvinsh L., Egorova M. (2022). Exosomes Released by Influenza-Virus-Infected Cells Carry Factors Capable of Suppressing Immune Defense Genes in Naive Cells. Viruses.

[64] Jiang Y., Cai X., Yao J., Guo H., Yin L., Leung W., Xu C. (2020). Role of Extracellular Vesicles in Influenza Virus Infection. Front. Cell. Infect. Microbiol..

[65] Zeitler M., Steringer J.P., Muller H.M., Mayer M.P., Nickel W. (2015). HIV-Tat Protein Forms Phosphoinositide-dependent Membrane Pores Implicated in Unconventional Protein Secretion. J. Biol. Chem..

