# Supplementary material for: TRIM21 Neutralizes Hazara Virus Using a Dual Mechanism of Nucleoprotein Caging and Ubiquitination
Source: Viruses. 2026 Sep 4;18(9):974. doi: 10.3390/v18090974 (PMC13611833; doi:10.3390/v18090974)
Supplement: Supplementary file 1 [file viruses-18-00974-s001.zip › supplementary Table S1.pdf]

Supplementary Table S1: List of primers and oligo standards used

sn	**Target**	**Sequence**	**Ref.**
1.	Standard S segment qPCR primers	-FWD: GTCGACCAAGACAAGGCAGA<br>-REV: GGGACACAGATGGGAAGGTG	This work
2.	-veRNA RT primer	GGCTCTGGGCGCTGGAAATGAGAGGGACGC CATCTACA	This work
3.	+veRNA RT primer	GCTGAACGCCGTTAGCTATCACCTTCACGGT GTCAGAGTC	This work
4.	-veRNA qPCR primers	-Virus-specific: ACCTTCACGGTGTCAGAGTC<br>-Non-viral tag: GGCTCTGGGCGCTGGAAA	This work
5.	+veRNA qPCR primers	-Virus-specific: TGAGAGGGACGCCATCTACA<br>-Non-viral tag: GCTGAACGCCGTTAGCTATC	This work
6.	-veRNA standard	GGCTCTGGGCGCTGGAaatgagagggacgccatctac agctcagcactcattgaggccacccgattctgtgctcctatcatgga gtgtgcatgggcttcctgcactgggacagttaaaagaggtctggaa tggttcgacaaaaacaaagactctgacaccgtgaaggt	This work
7.	+veRNA standard	GCTGAACGCCGTTAGCTATCaccttcacggtgtcaga gtctttgtttttgtcgaaccattccagacctcttttaactgtcccagtgca ggaagcccatgcacactccatgataggagcacagaatcgggtgg cctcaatgagtgctgagctgtagatggcgtccctctca	This work
