# Supplementary material for: TRIM21 Neutralizes Hazara Virus Using a Dual Mechanism of Nucleoprotein Caging and Ubiquitination
Source: Viruses. 2026 Sep 4;18(9):974. doi: 10.3390/v18090974 (PMC13611833; doi:10.3390/v18090974)
Supplement: Supplementary file 1 [file viruses-18-00974-s001.zip › supplementary Table S2.pdf]

Supplementary Table S2: List of plasmids used

sn	Name	Insert/description	Source	Comments
1.	pMK-RQ-S-EGFP	S segment rescue plasmid, EGFP reporter	Fuller *et al.* 2019	For generating recombinant HAZV, encoding EGFP reporter, WT NP
2.	pMK-RQ-S-mScarlet	S segment rescue plasmid, mScarlet reporter	This work	For generating recombinant HAZV, encoding mScarlet reporter, WT NP
3.	pMK-RQ-S-mScarlet_N-HA	S segment rescue plasmid, mScarlet reporter	This work	For generating recombinant HAZV, encoding mScarlet reporter, HA-tagged NP
4.	pMK-RQ-S-mScarlet_N-ALFA	S segment rescue plasmid, mScarlet reporter	This work	For generating recombinant HAZV, encoding mScarlet reporter, ALFA-tagged NP
5.	pMK-RQ-M	M segment rescue plasmid	Fuller *et al.* 2019	For generating recombinant HAZV
6.	pMK-RQ-L	L segment rescue plasmid	Fuller *et al.* 2019	For generating recombinant HAZV
7.	pCAG-T7pol	T7 polymerase	Addgene #59926	
8.	p874 T21MPP	Empty vector (EV)	*Zeng et. al.* 2021	Lentiviral vector for generating TRIM21-KO HeLa+EV, expression driven by native TRIM21 promoter
9.	p930 T21MPP	WT TRIM21	*Zeng et. al.* 2021	Lentiviral vector for generating TRIM21-KO HeLa+TRIM21^{WT}, expression driven by native TRIM21 promoter
10.	p894 T21MPP	TRIM21$^{M10E/M72E}$	*Zeng et. al.* 2021	Lentiviral vector for generating TRIM21-KO HeLa+TRIM21$^{M10E/M72E}$, expression driven by native TRIM21 promoter
11.	p915 T21MPP	TRIM21^{D355A}	*Zeng et. al.* 2021	Lentiviral vector for generating TRIM21-KO HeLa+TRIM21^{D355A}, expression driven by native TRIM21 promoter
12.	p916 T21MPP	TRIM21^{R55A}	*Zeng et. al.* 2021	Lentiviral vector for generating TRIM21-KO HeLa+TRIM21^{R55A}, expression driven by native TRIM21 promoter
13.	p928 T21MPP	TRIM21$^{\Delta RB}$	*Zeng et. al.* 2021	Lentiviral vector for generating TRIM21-KO HeLa+TRIM21$^{\Delta RB}$, expression driven by native TRIM21 promoter
