# Supplementary material for: TRIM21 Neutralizes Hazara Virus Using a Dual Mechanism of Nucleoprotein Caging and Ubiquitination
Source: Viruses. 2026 Sep 4;18(9):974. doi: 10.3390/v18090974 (PMC13611833; doi:10.3390/v18090974)
Supplement: Supplementary file 1 [file viruses-18-00974-s001.zip › supplementary Table S3.pdf]

Supplementary Table S3: List of antibodies used

sn	Target	Purpose	Supplier	Catalogue #	Dilution
1.	Mouse anti-GAPDH	Western blot	Ambion	AM4300	1:5000
2.	Rabbit anti-HA	Western blot	Sigma Aldrich	H6809	1:500
3.	Mouse anti-HA	Western blot	Enzo	ENZ-ABS118	1:500
4.	Rabbit anti-ALFA	Western blot	NanoTag	N1580	1:500
5.	Mouse anti-TRIM21 (D12)	Western blot	Santa Cruz	sc-25351	1:200
6.	Sheep anti-HAZV NP serum	Western blot	Dr John Barr	-	1:5000
7.	Sheep anti-HAZV NP serum	Split-luciferase assay	Dr John Barr	-	-
8.	Anti-HA (12CA5, human Fc) WT	EDNA	Genscript	-	-
9.	Anti-HA (12CA5, human Fc) H433A	EDNA	Genscript	-	-
10.	Anti-HA (12CA5, rabbit Fc) WT	EDNA	Genscript	-	-
11.	NbALFA-Fc (human Fc) WT	EDNA	Genscript	-	-
12.	NbALFA-Fc (human Fc) H433A	EDNA	Genscript	-	-
13.	Affinipure mouse anti-rabbit	EDNA	Jackson labs	211-005-109	-
14.	Affinipure rabbit anti-mouse	EDNA	Jackson labs	315-005-045	-
15.	Rabbit anti-HAZV NP serum	EDNA	Non-commercial	-	-
16.	Rabbit anti-HAZV NP serum	Confocal (1°)	Non-commercial	-	1:1000
17.	Rabbit anti-HA (C29F4)	Confocal (1°)	Cell signalling	#3724	1:400
18.	Mouse anti-HAZV NP serum	Confocal (1°)	Non-commercial	-	1:1000
19.	Rabbit anti-ALFA	Confocal (1°)	NanoTag	N1580	1:400
20.	Mouse anti-polyubiquitin FK2	Confocal (1°)	Enzo	BML-PW8810	1:400
